# Diagnosis of Acute Pulmonary Embolism on a Non-contrast CT Scan

**DOI:** 10.5334/jbsr.3295

**Published:** 2023-11-22

**Authors:** Letizia Ebser, Emmanuel Coche

**Affiliations:** 1 Department of Radiology, Cliniques Universitaires Saint-Luc, UCLouvain, BE

**Keywords:** acute pulmonary embolism, halo sign, non-contrast chest CT-scan

## Abstract

**Teaching Point:** There are two important signs that could be seen on a non-contrast chest CT scan that can lead to the diagnosis of a pulmonary embolism: the hyperdense pulmonary artery sign and pulmonary infarction.

## Case History

**Figure 1:**
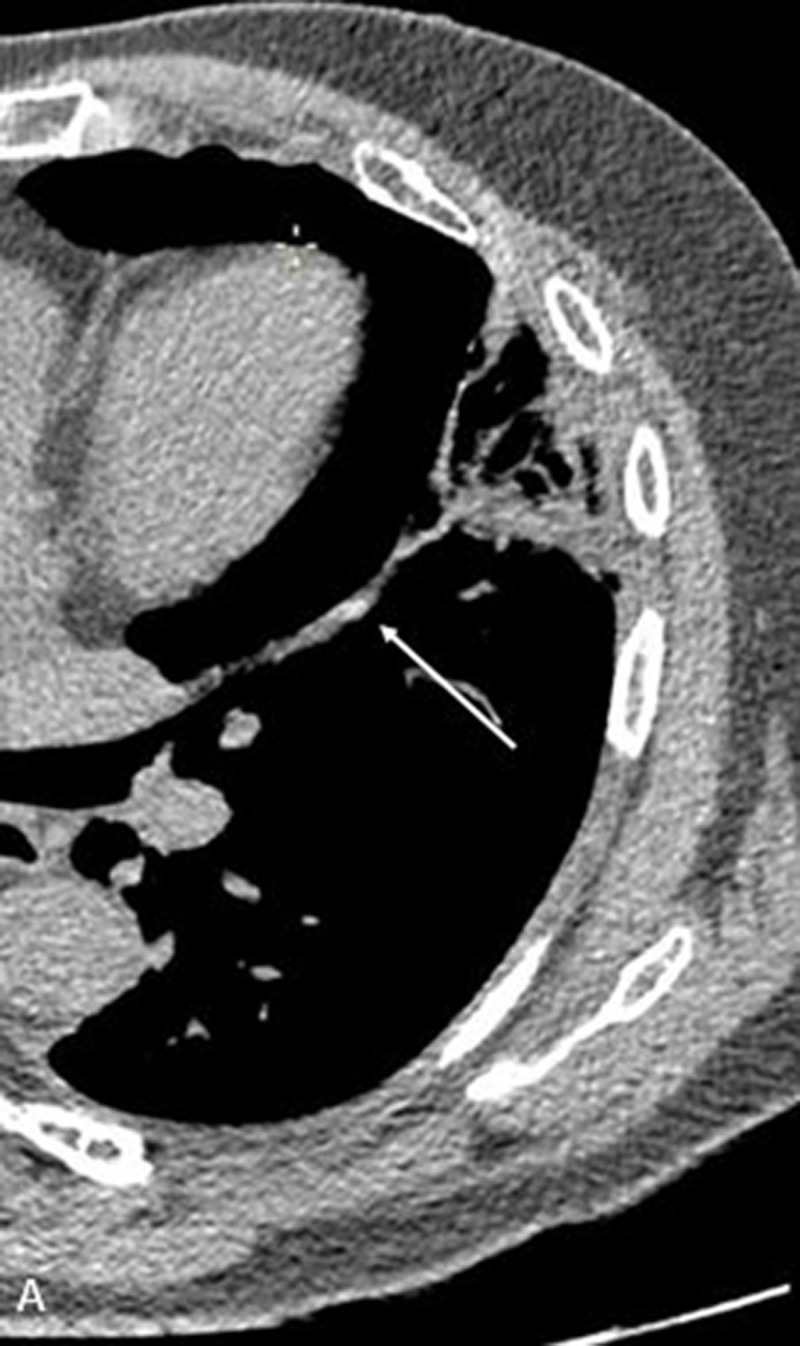


A 61-year-old male patient was referred to the emergency department for dyspnea (grade 3 classification, NHYA 2) for one week. Blood analysis showed C-reactive protein (CRP) at 138 mg/L and D-dimers at 1506 ng/mL. The patient was known to have an allergy to iodinated contrast medium. During a previous computed tomography (CT) scan with injection of iodinated contrast medium, the patient developed a severe hypersensitivity reaction. The hypersensitivity reaction signs that were reported were tremors, which led to syncope with signs of shock. The chest X-ray was normal, and the Doppler ultrasound (US) of the lower limbs was negative. A thoracic CT scan without contrast injection was therefore performed and revealed a spontaneously hyperdense subsegmental pulmonary artery in the lingula (Figure 1, axial plane; the white arrow depicts the hyperdensity within the segmental pulmonary artery). The density measured in the vessel involved by pulmonary embolism was 210 UH compared to the density in the main pulmonary artery that was measured at 55 UH.

**Figure 2:**
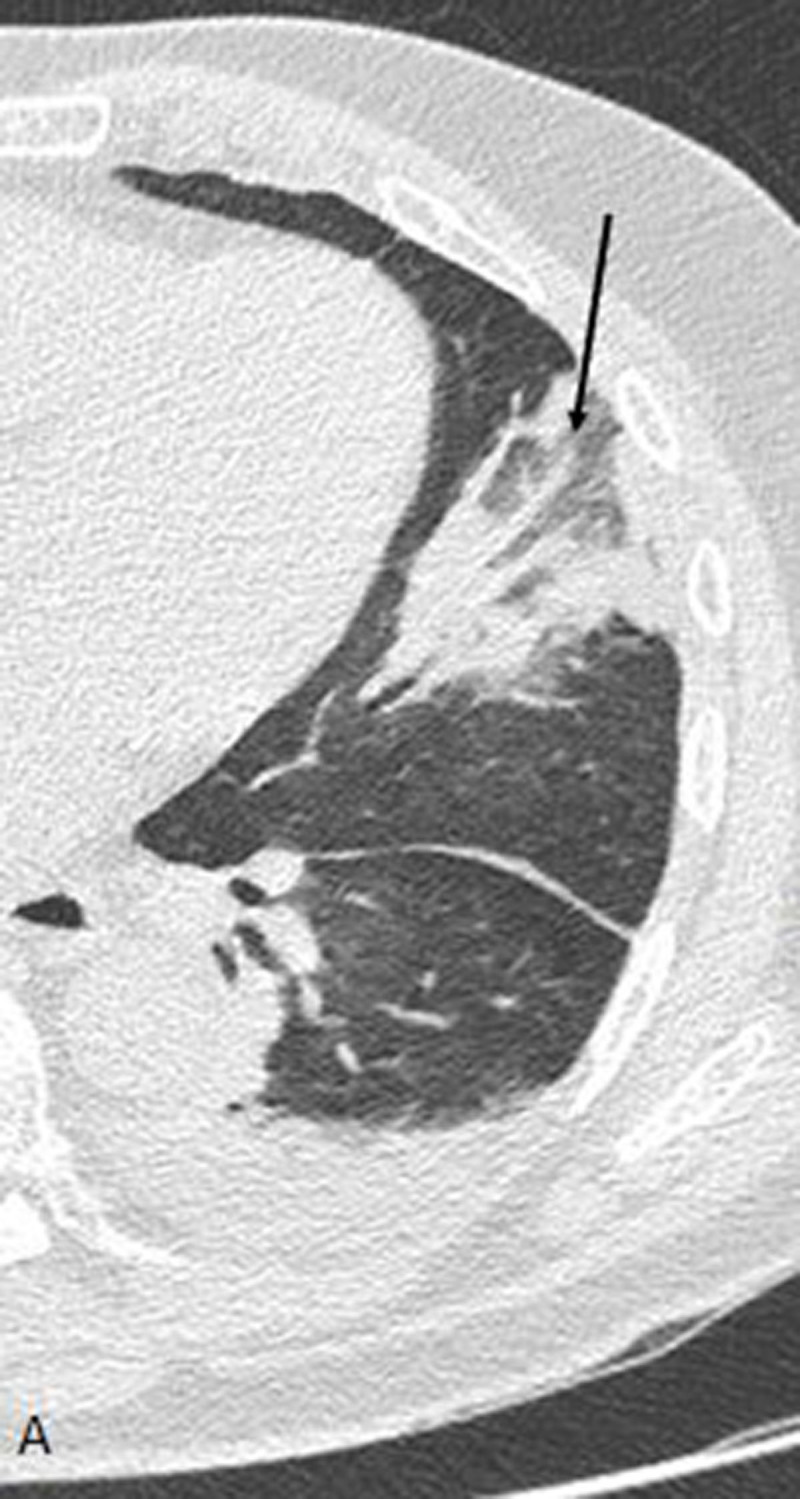


The artery was connected to a triangular sub-pleural consolidation with a central area of ground glass opacity surrounded by an air-space consolidation with the shape of a ring (such in this case) or a crescent (Figure 2, axial plane; the white arrow depicts the wedge-shaped opacity with reverse ‘halo sign’). Moreover, this sign has been associated with several different pathologies; therefore, those mentioned are but a few examples, and that should be acknowledged. It can also be observed in invasive fungal pneumonia, vasculitides, and cryptogenic organizing pneumonia (COP).

**Figure 3:**
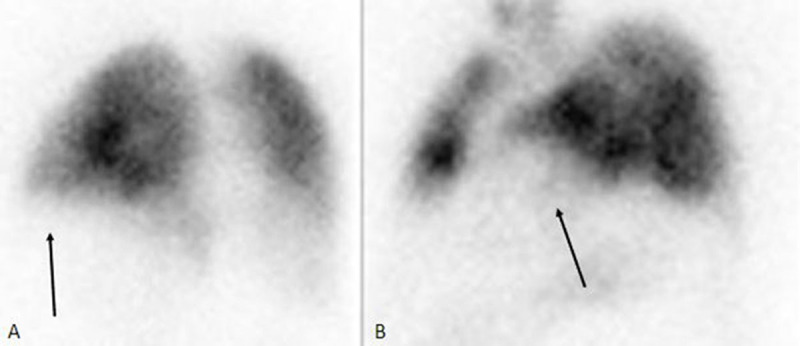


The combination of these CT findings was highly suggestive of an acute peripheral pulmonary embolism and was confirmed by pulmonary scintigraphy (Figure 3, oblique posterior left view (A) and oblique anterior left view (B); the white arrow shows a perfusion defect in the sub-segmental left inferior lingula artery territory).

A lung scintigraphy including only perfusion (Q) sequences, after injection of 4.5 cCi of MAA-Tc99m was realized. Six incidences were performed. In two incidences there was a perfusion defect in the territory of the sub-segmental inferior lingula artery that corresponded exactly to the pulmonary infarction seen on the thoracic CT scan. A diagnosis of pulmonary embolism was made based on PISAPED criteria.

The patient was treated with Rivaroxaban (Xarelto) 15 mg x 2 a day for 21 days and oxygen therapy. The follow-up with the patient showed a good clinical evolution.

## Comments

The gold standard for diagnosing acute pulmonary embolism is CT pulmonary angiography. In the context of renal failure, allergy to iodinated contrast agents or unavailability of other techniques such as lung scintigraphy or magnetic resonance imaging (MRI), a non-contrast CT could be performed to find CT arguments in favor of acute pulmonary embolism or alternative diagnoses.

Our case illustrates two facets of the thromboembolic disorder. First, the hyperdense pulmonary artery sign. This sign is very specific, and its diagnostic accuracy is approximately 80% in most recent studies [1]. The sensitivity depends on the localization of the clot. In fact, a central embolism has a sensitivity approaching 65% while the sensitivity for a peripherical embolism is 20% [1]. The spontaneous hyperdensity in the pulmonary artery lumen is due to the high concentration of erythrocytes in the clot compared to the adjacent blood flow. Differential diagnosis includes embolism of foreign material, such as talcosis or cement migration, after percutaneous vertebroplasty. Second, the wedge-shaped subpleural opacity with ‘reverse halo’ appearance, suggestive for pulmonary infarction. The central area of ground glass (possibly containing internal lucencies due to the presence of aerated alveoli in the infarcted lung tissue) represents the central core of the infarct, while the peripheral consolidation corresponds to histopathological findings such as inflammatory reaction, hemorrage, and organized thrombi.
